# Tadalafil attenuates ischemic damage as well as reperfusion injury in the rat ovary

**DOI:** 10.4274/jtgga.galenos.2019.2018.0121

**Published:** 2020-03-06

**Authors:** Çağdaş Şahin, Nuri Yıldırım, İsmet Hortu, Ali Akdemir, Serdar Özşener, Gürkan Yiğittürk, Oytun Erbaş

**Affiliations:** 1 Department of Obstetrics and Gynecology, Ege University Faculty of Medicine, İzmir, Turkey; 2 Department of Histology and Embryology, Muğla Sıtkı Koçman University Faculty of Medicine, Muğla, Turkey; 3 Department of Physiology, Demiroğlu Bilim University Faculty of Medicine, İstanbul, Turkey

**Keywords:** Phosphodiesterase type-5, tadalafil, ischemia-reperfusion injury, vasodilatation; anti-oxidant

## Abstract

**Objective:**

Tadalafil is a selective phosphodiesterase type-5 inhibitor with a long half-life. It has a dual function in ischaemic and re-perfused tissues, i.e. vasodilatation and anti-oxidant effects. These features of tadalafil distinguish it from other anti-oxidants. We investigated the dual effect of tadalafil on ischaemia and reperfusion injury in the rat ovary.

**Material and Methods:**

We established five study groups. Group 1 (n=6): sham-operated; group 2 (n=6): torsion; group 3 (n=6): torsion and Tadalafil; group 4 (n=6): torsion/de-torsion; and group 5 (n=6): torsion/de-torsion and tadalafil. Ovarian samples were harvested from animals and evaluated in terms of histopathologic changes, tissue malondialdehyde (MDA) concentrations, lactate production, and plasma cyclic guanosine monophosphate (cGMP).

**Results:**

Follicular degeneration, oedema, haemorrhage, and inflammatory cells were significantly decreased in group 5 in comparison with group 4. Group 2 and group 3 were compared in terms of vascular congestion and haemorrhage; these parameters were significantly decreased in group 3. In addition, significantly decreased MDA and lactate concentrations were observed in group 5 in comparison with group 4. Increased cGMP concentrations were detected in group 3 and group 5.

**Conclusion:**

We conclude that tadalafil might be useful in protecting the ovary against ischaemia and reperfusion injury. In the evet of ovarian torsion, it will provide a greater therapeutic effect than only performing de-torsion of the ovary or using other anti-oxidant agents.

## Introduction

Ovarian torsion is an emergency condition in gynaecology practice. It is particularly important when diagnosed in the reproductive period. Ischemia-reperfusion (IR) injury can damage ovarian tissue and also reduce the ovarian reserve (1). The time of diagnosis is important due to the diminishing ovarian reserve. However, it usually takes some time to achieve a diagnosis given the non-specific diagnostic symptoms. In addition, although restoration of the blood supply of ovarian tissue can reduce ischemic damage, it can also cause reperfusion injury.

When the ischemic period is extended, the associated cell damage becomes irreversible. During the ischemic stage, the production of adenosine triphosphate (ATP) decreases and anaerobic glycolysis begins. Decreasing ATP concentrations leads to the cessation of sodium-potassium pump channel function and subsequent water influx into cells, resulting in cell swelling. If ischemia persists, cells proceed to an irreversible stage. At this stage, severe swelling is seen in the mitochondria, as well as cell membrane damage. Cell death usually results in necrosis.

Reinstating the blood supply of ischemic tissue leads to recovery of cells in the reversible stage. However, this situation brings another problematic condition, reperfusion damage. The reoxygenation of ischemic tissues results in the production of reactive oxygen species (ROS), including superoxide anions (O2-), hydrogen peroxide, and hydroxyl radicals (OH-), among others. These ROS damage phospholipids and proteins of the cell membrane and promote mitochondrial permeability which can cause a reduction in ATP and lead to cell death (2). At this stage, the use of antioxidant agents helps to support the self-antioxidant defence mechanism of cells.

One of the phosphodiesterase type-5 (PDE-5) inhibitors is tadalafil which has been used in the treatment of erectile dysfunction (3). As PDE-5 catalyses the hydrolysis of 3'5'-cyclic guanosine monophosphate (cGMP), PDE-5 inhibitors cause an increase in cGMP. Nitric oxide (NO) is a potent vasodilator, and works via the secondary messenger cGMP. PDE-5 inhibitors facilitate the accumulation of cGMP within cells, and also facilitate NO-mediated vasodilation of vascular smooth muscle in mammals. Recent studies have demonstrated a potential beneficial effect of PDE-5 inhibitors on IR injury in the brain (4), kidney (5), and heart (6). These authors found that increasing cGMP attenuates lipid peroxidation (7) and nicotinamide-adenine dinucleotide phosphate [NAD(P)H] oxidase activity, which are the main sources of ROS production in oxidative stress (6).

Tadalafil has a dual function as a vasodilator and antioxidant. These characteristics distinguish it from other antioxidants used to prevent IR injury. In the current study, we investigated the effect of tadalafil on the ischemic stage and on reperfusion injury.

## Material and Methods

### Animals

Thirty mature, female Sprague-Dawley albino rats were used in this study. All rats were aged 12 weeks and weighed 200-220 g. Animals were housed in steel cages maintained in a temperature-controlled room (21±1 °C) under a 12-h light/dark cycle and fed ad libitum. This study was approved by the intuitional animal care committee, and all procedures were performed according to the experimental guidelines of Ege University (SPK-HADYEK 34562/2015/24). Patient approval has not been obtained as it is performed on animals. The oestrous stage was determined by taking a vaginal smear from all animals, and cell types were identified under a microscope using the Papanicolaou staining procedure. Oestrous was confirmed in the smear specimens from 30 rats, which were included in the experiment.

### Experimental design

Animals were anaesthetised with 50 mg/kg ketamine and 7 mg/kg xylazine hydrochloride (Alfasan Int. BV, the Netherlands). The skin of the abdominal area was trimmed and cleaned with povidone iodine. A 2 cm midline incision was performed on the abdomen, and the uterus and ovaries were detected.

In the sham-surgery group (group 1, n=6), laparotomy was performed and the abdomen was closed 1 min later without performing any surgical procedures. In the torsion group (group 2, n=6), ischemia was created for 3 h by applying atraumatic vascular clips to the vascular pedicles of ovaries on both sides. The incision was subsequently closed with 3/0 silk sutures. In the torsion and tadalafil group (group 3, n=6), ischemia was performed, as described for group 2, 30 min after administering 20 mg/kg of tadalafil [(Cialis, Lilly, IN, United States of America (USA)] via oral gavage. In the torsion/detorsion group (group 4, n=6), 3 h of ischemia was created, as in group 2, followed by 3 h of reperfusion. In the torsion/detorsion and tadalafil group (group 5, n=6), torsion was created for 3 h, followed by tadalafil administration 30 min prior to 3 h of detorsion/reperfusion. At the end of the reperfusion stage, both ovaries were harvested for histologic and biochemical evaluation.

### Histopathologic evaluation of tissue samples

Ovarian samples were evaluated using a light microscope. Specimens were fixed in 10% buffered formalin, then an increasing alcohol series was used to dehydrate samples. Subsequently, samples were cleared with xylene and embedded in paraffin. Tissue sections were sliced at 4-µm thickness and slides were stained with haematoxylin and eosin (H&E) prior to histologic analysis. An Olympus BX51 microscope connected to an Olympus C-5050 digital camera (Olympus Corp., Tokyo, Japan) was used for the analysis and photography of sections. Histologic sections were evaluated in terms of primordial and developing follicles. In the histology of the ovary, primordial follicles are located beneath of the cortex, surrounded by a single layer of granulosa cells. Primary follicles are encircled by a single layer of cuboidal granulosa cells. The identification of stratified granulosa cells indicates secondary follicles, and stratum granulosum and antral space are major features of tertiary follicles. The pyknotic appearance of the nucleus and collapsed ooplasm, with or without irregular granulosa cells, indicate a degenerated follicle.

The rate of vascular congestion, stromal haemorrhage and oedema, follicular degeneration, and infiltration of inflammatory cells in sections for each animal were scored from 0 to 3 according to the severity of injury. A score of 0 represents normal histology, and <33%, 33-66%, and >66% pathologic findings in the ovarian sections were scored as 1, 2, and 3, respectively (8).

### Determination of lipid peroxidation and total protein concentrations

Tissue samples were homogenised in KCl (150 mM) and centrifuged at 5000 g for 10 min. Lipid peroxidation was determined by analysis of the supernatant, with malondialdehyde (MDA) concentrations measured as thiobarbituric acid-reactive substances in each tissue sample (9). Trichloroacetic acid and thiobarbituric acid reactive substances reagents were mixed with tissue samples and incubated at 100 °C for 60 min. The samples were centrifuged at 3000 rpm for 20 min after cooling on ice, and the absorbance of the supernatant was read at 535 nm. The standard calibration curve obtained using tetraethoxypropane was used to calculate tissue MDA concentrations, which were expressed as nmol/µg protein. Bradford’s method was used to determine the total protein level in the tissue samples, with bovine serum albumin used as the standard (10).

### Measurement of plasma cGMP and lactic acid concentrations

After administering tadalafil, plasma samples were collected and stored at -80 °C until use for the cyclic nucleotide assay. Enzyme-linked immunosorbent assay (Cusabio, Biotech Co. Ltd. Wuhan, China) was used to determine the plasma cGMP level. The ultraviolet detection method was used to measure the L-lactic acid concentrations in plasma samples.

### Statistical analysis

Mann-Whitney U-test was used to compare the biochemical data and histopathologic scores between groups. The results were considered statistically signiﬁcant if the p-value was <0.05. Statistical analyses were performed using SPSS software version 16.0 (Chicago, IL, USA).

## Results

### Histopathologic results

**Figure 1:**
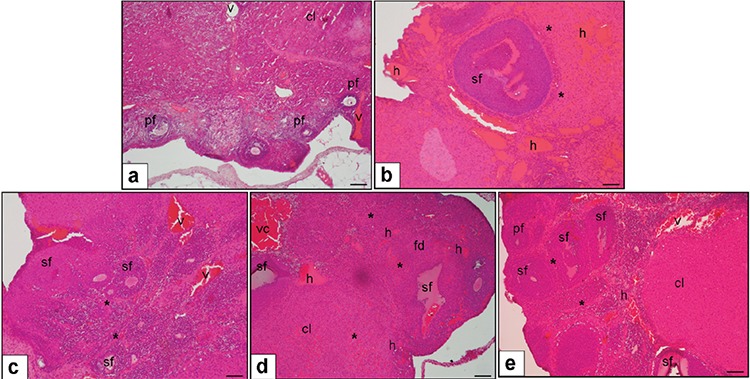
a. No pathologic changes were detected in the sham-operated animals. (v) vessel, (sf) secondary follicle, (pf) primary follicles, (cl) corpus luteum. b. Haemorrhaging (h) and oedema (*) were detected in the 3-h torsion group. c. Decreased vascular congestion (vc), and Haemorrhaging (h) were observed in the torsion and tadalafil group. d. Vascular congestion (vc), haemorrhaging (h), and oedema (*) were observed in the torsion/de-torsion group. e. Decreased oedema (*) and haemorrhaging were detected in the torsion/de-torsion tadalafil group. Haematoxylin and eosin staining was performed. Scale bars represent 250 μm

The histopathologic results of ovarian tissue samples of the five groups are presented in Figure 1, with no histopathologic changes observed in the group that received sham surgery (group 1). In contrast, increased follicular degeneration (p<0.05), oedema (p<0.05), vascular congestion (p<0.001), haemorrhage (p<0.05), and infiltration of inflammatory cells (p<0.05) were observed in the torsion group (group 2). When tadalafil was administered before torsion, a statistically significant decrease in vascular congestion (p<0.05) and haemorrhage (p<0.05) was found for group 3 compared with the torsion-only group (group 2).

When we evaluated the reperfusion effect, severe tissue damage was observed after restoration of the ovarian blood supply in group 4. Follicular degeneration and infiltration of inflammatory cells were more prominent in the torsion/detorsion-only group (group 4), and all abnormal findings were significantly decreased after adding tadalafil (group 5) when compared with group 4. A comparison of scores between groups in terms of histopathologic abnormalities is shown in Table 1.

### Biochemical changes

Increased MDA concentrations were found in the torsion (group 2) and torsion/detorsion (group 4) groups compared with the sham-surgery group (group 1, p<0.01). Decreased MDA concentrations were measured in the torsion/tadalafil (group 3) and torsion/detorsion/tadalafil (group 5) groups, but this decrease only reached statistical significance in the torsion/detorsion/tadalafil group (group 5, p<0.001). Similar findings were also observed for the lactate results. Increased tissue lactate concentrations were found in the torsion (group 2) and torsion/detorsion (group 4) groups. Importantly, lactate concentrations were normalised in the groups that received tadalafil (groups 3 and 5). Plasma cGMP concentrations reflected the effect of tadalafil treatment, and a statistically significant increase in cGMP concentrations was found in the groups that were administered tadalafil (groups 3 and 5, p<0.01). The biochemical results are presented in Table 2.

## Discussion

The aim in cases of ovarian torsion is to reduce the extent of necrotic tissue and preserve ovarian function. For this purpose, the ischemic period should be shortened and reperfusion injury should be prevented. Many antioxidant drugs have been investigated for their efficiency in preventing oxidative damage during the reperfusion period (11,12). One of them, tadalafil, has antioxidant activity, and attenuates the generation of ROS under oxidative stress (6). In addition, tadalafil also possess a vasodilation effect, leading to an increase in cGMP concentrations. These functions provide a valuable effect during the ischemic period of certain organs, such as the heart and kidney (13,14). In the current study, we demonstrated decreased vascular congestion and haemorrhage in ischemic ovaries with the use of tadalafil. Our findings are similar to those obtained by Lledo-Garcia et al. (15), who studied the use of sildenafil for renal ischemia and reported the effectiveness of administering sildenafil in warm ischemic kidneys during the immediate post-transplant period. The effectiveness of a PDE-5 inhibitor in ischemic ovaries was also revealed in our results.

Tadalafil has a similar function to other PDE-5 inhibitors like sildenafil; however, it is more selective than sildenafil for the binding of PDE-5 (16) and has a longer half-life (17.5 vs 4.5 h, respectively) (17). These features mean that tadalafil is longer acting. Tadalafil augments the effect of NO by decreasing cGMP degradation. NO plays a major role in inhibiting NAD(P)H oxidase and limiting neutrophil and platelet adhesion, aggregation, and activation (18).

Increased tissue MDA indicates lipid peroxidation because MDA concentrations increase under conditions of oxidative stress (19). MDA impairs the ionic transport and enzymatic activity of cells, causing major changes in cell membrane permeability, resulting in damage and breakages that separate cell and organelle contents (20). In the event of ischemia, energy production is interrupted, which reduces the concentration of ATP in cells. These cells begin anaerobic energy production, increasing the lactic acid content and decreasing the pH level of cells. Arikan et al. (21) previously investigated the effect of tadalafil on IR injury in rat ovaries, and demonstrated decreased MDA concentrations and increased catalase (CAT) and superoxide dismutase (SOD) activities, which are scavenger enzymes for ROS, after using tadalafil (21). Additionally, they observed reduced histopathologic damage in groups that received tadalafil, which led the authors to conclude that tadalafil had a protective effect against IR injury. Similarly, increased MDA and lactate concentrations were ameliorated by tadalafil in our study, and these biochemical findings are supported by the histopathologic results of the tadalafil groups.

Another PDE-5 inhibitor, sildenafil, has also been investigated in ovarian IR injury. The authors found changes in MDA, myeloperoxidase (MPO), SOD, glutathione peroxidase (antioxidant enzyme) between treated and untreated groups (22). MPO is secreted by neutrophils and is used as a marker of neutrophil activation in IR injury. These authors observed decreased MDA and MPO concentrations and increased tissue antioxidant enzyme concentrations, as well as an improved histologic appearance, in groups treated with sildenafil (22).

The effectiveness of PDE-5 inhibitors on reperfusion injury in ovaries has been confirmed by two studies (21,22). The investigators demonstrated an antioxidant effect of PDE-5 inhibitors on reperfusion injury. The vasodilator feature of PDE-5 inhibitors also contributes to this effect. Therefore, in the current study, we investigated the vasodilator effect of tadalafil during the ischemic stage and found attenuated vascular damage in the ischemia groups that were administered tadalafil. These findings indicate that tadalafil has a dual function to reduce ischemia and reperfusion damage in the ovary.

A limitation of this study is the absence of long-term observations for both the biochemical and histologic parameters. Thus, future long-term studies should be performed to determine whether the changes in histologic and biochemical parameters are transient or permanent.

The current management of ovarian torsion includes the protection of ovaries after detorsion, and avoids oophorectomy where possible (23). In this situation, prophylactic measures against injury are implemented, which affect ovarian function (24). Sometimes, the diagnosis of ovarian torsion takes time due to its non-specific symptoms. However, prompt diagnosis of ovarian torsion is important to preserve ovarian function. Therefore, the use of tadalafil in patients with suspected ovarian torsion will provide a greater therapeutic benefit than if treatment is only started following ovary detorsion. This treatment resembles the use of acetylsalicylic acid in patients with suspected myocardial infarction in the ischemic stage.

## Figures and Tables

**Table 1:**
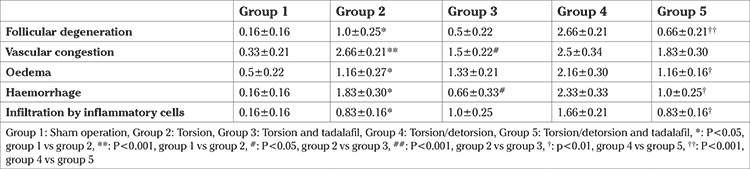
Histopathologic findings in the groups

**Table 2:**
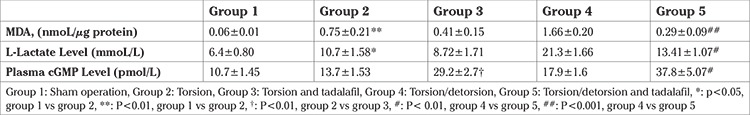
Tissue biochemical parameters in groups
